# Comparative Proteomic Profiling of Adrenocortical Neoplasia Using Mass Spectrometry

**DOI:** 10.1021/acs.jproteome.5c00740

**Published:** 2025-11-21

**Authors:** Jean Lucas Kremer, Henrique Sanchez Ortega, Talita Siqueira-Souza, Claudia Blanes Angeli, Leo Kei Iwai, Claudimara Ferini Pacicco Lotfi

**Affiliations:** † Institute of Biomedical Sciences, Department of Anatomy, 54544 University of São Paulo, Av. Prof. Lineu Prestes, 2415, Butantan, São Paulo 05508-000, Brazil; ‡ School of Medicine, Department of Clinical Medicine, University of São Paulo, Av. Dr. Arnaldo, 455, Cerqueira Cesar, Brazil 01246903 São Paulo; § Institute of Biomedical Sciences, Department of Parasitology, University of São Paulo, Av. Prof. Lineu Prestes, 1374, Butantan, São Paulo 05508-000, Brazil; ∥ Butantan Institute, Laboratory of Applied Toxicology, Center of Toxins, Immune-response and Cell Signaling LETA/CeTICS Laboratory, Av. Vital Brasil, 1500 - Butantã, São Paulo 05503-900, Brazil

**Keywords:** adrenal gland, adrenocortical neoplasia, proteome, bioinformatics

## Abstract

Recent advances in high-throughput molecular analysis have significantly enhanced our understanding of the molecular mechanisms underlying adrenocortical diseases. To identify differences in protein signatures that may reveal insights into disease-specific pathogenesis, we used LC–MS/MS and bioinformatics to compare proteomic profiles of normal human adrenal (NHA) tissue, adrenocortical adenomas (ACA), adrenocortical carcinomas (ACC), and primary macronodular adrenocortical hyperplasia (PMAH) tumors, with and without *ARMC5* mutations. In total, 7350 proteins were identified, and 3976 were quantified across all samples. Differentially expressed proteins (DEPs) were found in ACA vs NHA (27 DEPs), ACC vs NHA (49 DEPs), and PMAH vs NHA (81 DEPs). Comparing ACC and ACA revealed 64 upregulated and 48 downregulated DEPs. PMAH with *ARMC5* mutations (PMAHw) vs without *ARMC5* mutations (PMAHwt) had the fewest DEPs, 12 upregulated and 4 downregulated proteins in PMAHw. These findings were validated using an independent ACC cohort from Seoul National University Hospital, which showed 99.8% overall similarity and with no significant disparities. This comprehensive profiling of NHA, ACA, ACC, and PMAH offers insights into normal adrenal function and tumor-associated changes. Our study presents a high-quality proteomic data set, highlighting potential biomarkers and therapeutic targets, and makes a significant contribution to the understanding of adrenocortical disease mechanisms.

## Graphical abstract

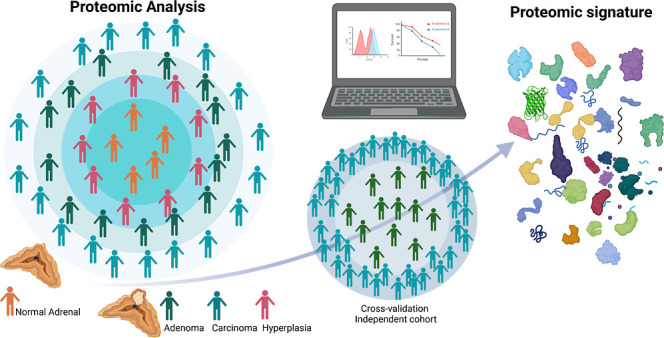

## Introduction

Adrenocortical nodular disease, adrenal cortical adenoma, and adrenal cortical carcinoma are recognized alterations of the adrenal cortex. A common condition among nodular diseases is sporadic adrenocortical hyperplasia. In contrast, micronodular and macronodular adrenocortical hyperplasia are rare conditions often associated with a germline pathogenic mutation.^1^ Primary macronodular adrenocortical hyperplasia (PMAH) is caused in nearly 50% of familial cases by pathogenic variants in *ARMC5* and, more rarely, by *KDM1A* inactivation, particularly in hereditary food-dependent Cushing’s syndrome.^2,3^ All are considered clonal benign proliferative conditions, with Cushing syndrome as a variable clinical manifestation depending on the level of cortisol secretion.^4^ Adrenocortical adenomas (ACA) are the most common tumors of the adrenal cortex, and when hormone-producing, they may involve the production of aldosterone or cortisol. Functional adenomas causing primary aldosteronism have somatic and, in some cases, germline mutations of several ion channel genes and activation of Wnt pathway effector beta-catenin (*CTNNB1*) that cause increased aldosterone production and cell proliferation.^5–7^ Functional adenomas that produce cortisol exhibit genetic alterations in the PKA pathway, whereas *CTNNB1* variants are the most frequent molecular alterations in nonfunctional adenomas, particularly in larger tumors.^8,9^ Adrenocortical carcinoma (ACC) is a rare disease (1–2 cases/million; 5 year survival rate of <60%) whose clinical presentation includes Cushing or virilization-feminization syndromes due to hormone production.^10^ According to the WHO 2022 classification system, adrenocortical carcinomas are categorized into morphological subtypes, including classical or conventional (97%), oncocytic (2%), myxoid (<1%), and sarcomatoid (<1%). This classification considers vascular invasion for both diagnostic and prognostic purposes, incorporating the mitotic count and *K*_i_-67 labeling index for risk stratification.^11^ Molecular studies of adrenocortical carcinomas have identified somatic variants, methylome, and microRNA expression profiles to classify, predict prognosis, and identify new therapeutic targets.^12,13^ Thus, our understanding of the molecular pathogenesis of adrenocortical diseases, particularly adrenocortical carcinomas, has made significant progress in recent years due to the application of high-throughput molecular analysis. Liquid chromatography-tandem mass spectrometry (LC–MS/MS) offers a method for identifying and quantifying a wide range of proteins in biological samples. It aims to enhance our comprehension of biological processes, reveal underlying mechanisms, and improve the diagnosis and prognosis of diseases.^14,15^ To elucidate disease-specific signatures and gain a deeper understanding of the distinct pathogenesis of adrenal cortex disorders, we conducted LC–MS/MS-based proteomic profiling in this study, comparing adrenocortical adenomas, conventional adrenocortical carcinomas, and bilateral macronodular adrenal disease samples with each other and with normal human adrenal tissue. To validate the results, an independent ACC cohort was used, which showed an overall average similarity of 99.8%.

## Experimental Section

### Normal Adrenal Glands and Patients

The normal human adrenal glands from two men and three women (NHA, *n* = 5) were obtained from cadavers from the Death Verification Service of the University of São Paulo, as described by Kremer et al. (2024).^15^ Samples of adrenocortical tumors and hyperplasia [Adrenocortical Adenoma (ACA, *n* = 8); Adrenocortical Carcinoma (ACC, *n* = 8); Primary Macronodular Adrenal Hyperplasia (PMAH, *n* = 10) were classified as PMAH with *ARMC5* pathogenic variants (PMAHw, *n* = 5); and PMAH without *ARMC5* mutation (PMAHwt, *n* = 5) obtained from Hospital das Clínicas at the School of Medicine of the University of São Paulo, responsible for evaluating and providing patient data. Table 1 and Figure S1 summarize the patients’ clinical and histological characteristics of normal and neoplasm fragments. Genetic information on ACC and PMAH patients, including germline pathogenic *TP53* and *ARMC5* variants, as well as hormone-producing status for ACC, is summarized in Table 2. In PMAHw of patients, germline variants of the *ARMC5* gene were identified in adrenal tissue, including a missense variant (patient 1), a nonsense variant (patients 2 and 4), a splice-site variant (patient 3), and a frameshift variant (patient 5). Second somatic mutation events were observed in the sample of patient 2, Loss of Heterozygosity (LOH), and an in-frame deletion in the sample of patient 3. Additional information on PMAH and *ARMC5* variants is also available in Cavalcante (2019).^16^

**Table 1:** Clinicopathological Characteristics of Patients^a^

characteristics	NHA (*n* = 5)	ACA (*n* = 8)	ACC (*n* = 8)	PMAH (*n* = 10)
age (year)				
median	65	53	44	61
range	31	42	35	22
Gender				
female/male ratio	3:2	8:0	2:6	1:9
weiss score (WS)	NA	2 ≤ 0	9 ≤ 4	NA
WS modified	NA	1 ≤ 0	7 ≤ 3	NA
Furlan nuclear grade	NA	3 ≤ 1	4 ≤ 3	NA

a NHA = Normal Human Adrenal Gland; ACA = Adrenocortical Adenoma; ACC = Adrenocortical Carcinoma; PMAH = Primary Macronodular Adrenal Hyperplasia; NA = Not Applicable.

**Table 2:** Genetic Information of ACC and PMAH Patients^a^

ACC	TP53 variant	hormone producing
1	c.818G > A (p.R273H) in heterozygosity (exon 8 – DNA-binding domain)	**+**
2	absence of pathogenic variants	**+**
3	c.1010G > A (p.R337H), heterozygous (exon 10 – tetramerization domain)	**+**
4	c.1010G > A (p.R337H), heterozygous (exon 10 – tetramerization domain)	**+**
5	absence of pathogenic variants	**+**
6	absence of pathogenic variants	**+**
7	absence of pathogenic variants	**+**
8	absence of pathogenic variants	**-**
PMAHw	*ARMC5* variant	
	germline	somatic
1	exon 3: c.968G > A, p.Gly323Asp	Absent
2	exon 3: c.1158G > A; p.Trp386*	LOH
3	exon 2: c.476 −1G > C	exon 6: c.2053_2055delCTT, p.del685Leu
4	exon 3: c.799C > T, p.Arg267*	Absent
5	exon 4: c.1094T > C, p.Leu365Pro	Absent

a ACC = adrenocortical carcinoma; PMAHw = Primary macronodular adrenal hyperplasia with *ARMC5* mutation; 1 = T52; 2 = T126; 3 = T127; 4 = T177; 5 = T191 (ref 16); LOH = Loss of Heterozygosity; + = hormone-producing, - = nonhormone-producing, absent = sequenced but not found.

The study was conducted in accordance with the ethical principles outlined in the Declaration of Helsinki. It was approved by the Ethics Committee of the Institute of Biomedical Sciences and the National Research Ethics Council (Comissão Nacional de Ética em Pesquisa—CONEP) with n° 6.524.377. Written informed consent was obtained from all patients or their legal guardians.

### Sample Preparation for Proteomic Analysis

After partially thawing, the normal adrenal gland was positioned on a frozen surface and cut in half along the sagittal plane. Sections of 3 mm thickness were then cut in the same plane, revealing all regions of the normal gland. Total proteins were extracted from sections of the normal adrenal gland, tumor, and hyperplasia fragments in an 8 M urea solution containing 5% protease inhibitors and incubated for 30 min at 56 °C until completely dissolved. The samples underwent sonication with a probe tip sonicator thrice on ice at 40% power for 30 s each. Protein extracts were quantified using Qubit (Thermo Fisher Scientific). The protein digestion and peptide purification were prepared according to the method outlined by Kawahara et al. (2021),^17^ with some modifications. Fifty micrograms of extracted proteins were treated with 10 mM DTT for 45 min at 30 °C, then alkylated with 40 mM iodoacetamide for 30 min at room temperature in the dark. The reaction was quenched by adding 5 mM DTT, and the samples were diluted to reach a 0.8 M urea concentration. The proteins were digested with trypsin (1:50, w/w; Promega) overnight at 37 °C. Samples were acidified with 1% trifluoroacetic acid (TFA) to halt trypsin digestion, then centrifuged at 10,000 rpm for 10 min to remove insoluble material. The resulting supernatant was collected and desalted using Stagetips containing a 3 M Empore C18 disk (Sigma-Aldrich) in a P200 tip. Lastly, the peptide samples were dried in a speed vacuum concentrator (Labconco, Kansas City, MO, USA).

### Liquid Chromatography Coupled to Tandem Mass Spectrometry Analysis

The analysis was performed as described by Kremer et al. (2024),^15^ with adaptations. The peptide mixture was analyzed using a spectrometer operated in Data-Independent Acquisition (DIA) mode, acquiring spectra in full MS mode with a resolution of 60,000 for MS1 determination. Mass range was set to 350–950 *m*/*z* with an isolation window of 10 *m*/*z*. The full MS AGC target was set to 300% with an injection time of 50 ms. AGC target value for fragment spectra was set to 1000% with an injection time of 50 ms. Sixty windows of 10 Da were acquired to cover the entire scan range. Normalized collision energy was set at 32% and 36%. The MS/MS spectra were acquired using two compensation voltages, −45 V and −60 V, in the FAIMS system, with a resolution of 30,000, a maximum injection time of 50 ms, a mass range of 120 to 2000 *m*/*z*, and a dynamic exclusion time of 45 s.

### Data and Bioinformatics Analysis

The raw files generated on the spectrometer were initially converted to mzML using the MSConvert software (SourceForge Inc.). Protein identification was performed using the DIANN program (version 1.9.1)^18^ against the *Homo sapiens* database downloaded from UniProt in June 2024. The search parameters used were as follows: trypsin as an enzyme allowing one missed cleavage, methionine oxidation as a variable modification, and carbamidomethylation of cysteine as a fixed modification; precursor *m*/*z* range of 350–950; peptide length range from 7 to 30; fragment ion *m*/*z* range of 200–1800; precursor charge range from 1 to 4; and precursor false discovery rate (FDR) set to 1%. The search was performed with a tolerance of 10 ppm for MS and 0.05 Da for MS/MS, and the proteomic data were analyzed using R Studio (version 4.3.3). Preprocessing steps included normalization and imputation of missing values. Missing values were imputed using the “pca” function from the R package “pcaMethods.” Log2 transformation was applied to the raw data, followed by Quantile normalization using the “normalize.quantiles” function from the “preprocessCore” package (2, 3). Differential expression analysis was performed using the “limma” package in R. Statistical significance was assessed by fitting a linear model to the normalized data and then applying empirical Bayes moderation of standard errors. Adjusted p-values were calculated using the Benjamini-Hochberg method to control the False Discovery Rate (FDR). Proteins with FDR values < 0.05 were considered statistically significant. The proteins were annotated using UniProtKB codes (https://www.uniprot.org/) and Gene Ontology AmiGO2 (https://amigo.geneontology.org/amigo/landing). Protein–protein interaction networks and functional enrichment analysis were performed using the STRING version 12.0 platform (https://string-db.org/) with a high-confidence interaction score (0.700), a similarity threshold of 0.8, and a false discovery rate (FDR) of 0.05. A summary was generated using the BioRender software (https://www.biorender.com/), accessed in September 2024. The mass spectrometry proteomics data have been deposited in the ProteomeXchange Consortium via PRIDE with the data set identifiers PXD062822 and 10.6019/PXD062822. The Supporting Information Tables contain data generated and cited throughout the manuscript.

### Assessment of Reproducibility and Cross-Validation of Proteomic Profiles from ACC-USP Using an Independent Cohort from SNUH

A quantitative comparison of our proteomic data with that from Seoul National University Hospital (SNUH) was performed using a stringent statistical protocol to confirm the reproducibility and alignment of our data set with an independent cohort.^19^ We filtered the data to include only proteins present in at least 35% of samples in each cohort, ensuring quality for further analysis. The data set was then normalized and imputed according to ACC_USP protocols, followed by batch effect correction to reduce technical variability.

A Global Similarity Index (GSI) was created to measure the similarity of proteomic profiles between two centers, scoring from 0 to 1. Higher values signify greater data set agreement. The GSI is a weighted average of five statistical measures: Pearson correlation (25%), intraclass correlation coefficient (ICC; 25%), percent similarity (20%), percent difference via Bland-Altman analysis (15%), and standardized Z-score (15%). A Bland-Altman analysis was performed to evaluate bias and variation in protein expression between data sets. Calculations included mean differences, limits of agreement, and Z-scores for each protein, revealing significant differences and confirming the concordance level. A stratified 4-fold cross-validation was employed to assess data robustness by partitioning the cohorts into four subsets for training and testing. This method evaluated correlation and stability for each protein and generated a composite score for consistent performance.

### Cross-Validation of Proteomic Data Through Clinical Correlation Analyses Using the GEPIA Platform

For this study, data corresponding to ACC and normal adrenal tissue were retrieved and analyzed. Clinical relevance was assessed using the GEPIA platform (Gene Expression Profiling Interactive Analysis; http://gepia.cancer-pku.cn), which provides interactive access to ACC-RNA sequencing data from the TCGA (The Cancer Genome Atlas) and GTEx (Genotype-Tissue Expression) projects. To evaluate the prognostic value of selected proteins, overall survival (OS) and disease-free survival (DFS) curves were generated. ACC samples from the TCGA and GTEx projects were stratified into high-expression (*n* = 38) and low-expression (*n* = 38) groups based on median RNA-seq expression levels. Statistical significance was determined using the log-rank test (*p* < 0.05 considered significant). The platform also provided hazard ratios (HRs), which indicate the relative risk of the event (death or recurrence) occurring in the high-expression group compared to the low-expression group. An HR > 1 suggests a worse prognosis (higher risk), while an HR < 1 indicates a better prognosis (lower risk) in the high-expression group. Clinical stage analysis enables the investigation of potential associations between gene expression levels and the progression of clinical disease. Gene expression levels were also analyzed based on the pathological staging of ACC (stages I–IV). Comparisons across stages were visualized using violin plots, and statistical significance was assessed using one-way analysis of variance (ANOVA), as implemented in the platform.

### Statistical Analysis

Statistical analysis was performed using R version 4.3.3. The p-value was corrected using the Benjamini-Hochberg method to control for the false discovery rate (FDR). Results are expressed as mean ± SD, and statistical significance was considered for *p* < 0.05.

## Results

### Quantitative Proteomic Analysis of Normal and Neoplasm Tissues

To identify the proteomic signatures of normal and neoplastic adrenal cortex, we analyzed normal human adrenal glands (*n* = 5), adrenocortical tumors (ACA = 8; ACC = 8), and primary macronodular hyperplasia of the adrenal cortex (PMHA = 10) fragments according to the experimental design presented in the workflow shown in Figure 1. Extracted proteins were prepared, purified, and analyzed using label-free quantitative liquid chromatography-tandem mass spectrometry (LC–MS/MS)- based proteomics. After raw files were processed and statistical analysis performed, total protein was identified and quantified, considering a minimum of 60% of valid values in at least one group. Protein–protein interaction networks and functional enrichment analysis were performed, and a student’s *t*-test (*p* < 0.05) was applied to identify differentially expressed proteins (DEPs) among normal and neoplasm samples. Comparative analysis of proteomic profiles between studies was conducted based on the results obtained (USP_ACC) at Seoul National University Hospital (SNUH_ACC).

**Figure 1:**
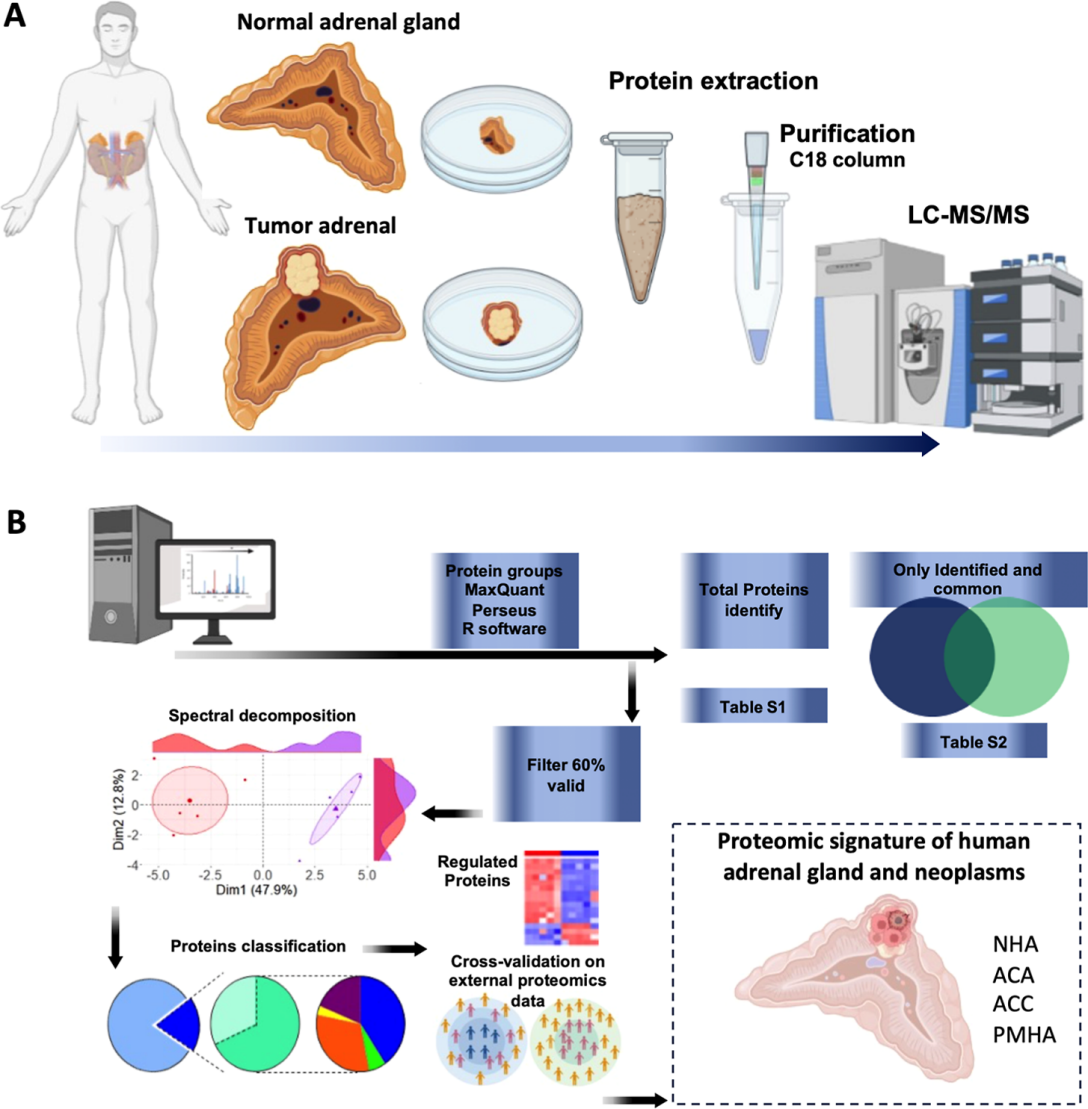
Overall workflow for the proteomic analysis of normal adrenal gland and neoplasm samples. (A) Workflow for processing normal human adrenal gland and neoplasm samples; (B) Workflow of bioinformatics tools for identifying, classifying, and validating proteins identified. This figure was created using Biorender.com (accessed in October 2024). LS-MS/MS stands for liquid chromatography-tandem mass spectrometry. USP_ACC = University of São Paulo and SNUH_ACC = Seoul National University Hospital cohorts.

### Protein Identification and Quantification in Normal and Neoplasm Adrenal Tissues

To confirm the adrenal tissue origin, we examined specific adrenal markers, including CYP11B1, CYP11B2, NR5A1, DAB2, WNT4, WNT2B, TH, and others (Table S1), to ensure an accurate representation of adrenal cortex tissue. A total of 7350 proteins were identified, 4646 proteins in NHA, 5531 in ACA, 6431 in ACC, 4687 in PMHAw, and 5199 in PMHAwt samples (Table S1), and 3976 proteins were quantified in all analyzed samples (Table S2, Figure 2A,B). Principal Component Analysis (PCA) using proteins categorized by tissue type indicated a distinct separation among samples (Figure 2C). The Venn diagram (Figure 2D) illustrates the identified proteins, exhibiting quantitative similarities and differences among the groups. Specifically, 50 proteins were identified in NHA, 83 proteins in ACA, 790 proteins in ACC, 25 proteins in PMAHwt, and 22 proteins in PMAHw (Table S3). In addition, protein groups (yellow dots) that are exclusive to each condition, as they are absent in any other sample groups analyzed, were identified: 17 proteins in NHA, 9 in ACA, 349 in ACC, 1 protein in PMAHw, and 1 in PMAHwt (Figure 2D; Table S3).

**Figure 2:**
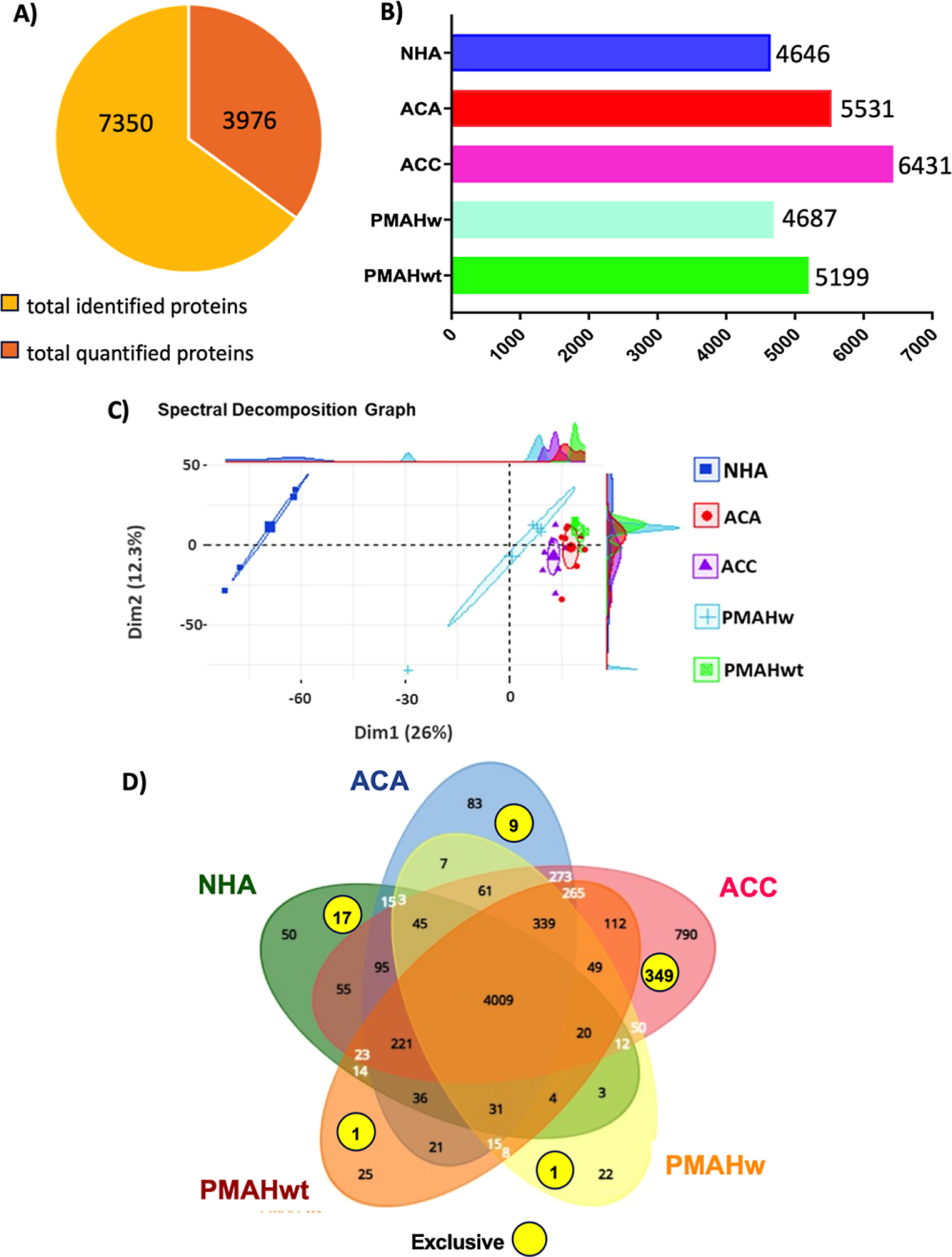
Proteins identified in normal and neoplasm adrenal samples. (A) Vein diagram showing the total and quantified proteins; (B) Number of proteins identified in different types of samples; (C) Principal Component Analysis (PCA) to show distinct separation among samples; (D) Vein diagram to illustrate the quantitative similarities and differences among the groups of identified proteins. NHA = normal human adrenal; ACA = adrenocortical adenoma; ACC = adrenocortical carcinoma; PMAH = primary macronodular adrenal hyperplasia with (w) and without (wt) *ARMC5* mutation.

### Differentially Expressed Protein (DEPs) of Normal Adrenal and Neoplasms

We compared the differential expression of multiple proteins to explore similarities and differences between the neoplasms and NHA (Table S4). In Figure 3A, the Venn diagram compares ACA and NHA, with 1064 proteins identified in ACA, 181 in NHA, and 4455 proteins commonly observed in both ACA and NHA. The heatmap (Figure 3B) and Volcano plots (Figure 3C) revealed statistically significant 27 DEPs between ACA and NHA, with 2 upregulated proteins, MT3 and RBM3, and 25 downregulated proteins, with NEFL, HAAO, SOD3, IGHA2, and HDGFL3 as the top five. In Figure 3D, we compared ACC and NHA with 1939 proteins identified in ACC, 156 in NHA, and 4480 proteins expressed in both groups. There were 49 DEPs between ACC and NHA, with 4 upregulated proteins DEFA3, NUP160, SRRM2, and RBM3, and 45 downregulated, with SOD3, CNRIP, DNAH1, GAMT, ADIRF, and CA3 as the top six downregulated proteins (Figure 3E,F). Correlation of proteomics-identified targets with clinical data in ACC using GEPIA revealed that SRRM2, NUP160, and RBM3 are associated with shorter OS, DFS, and advanced stage (Figure S2).

**Figure 3:**
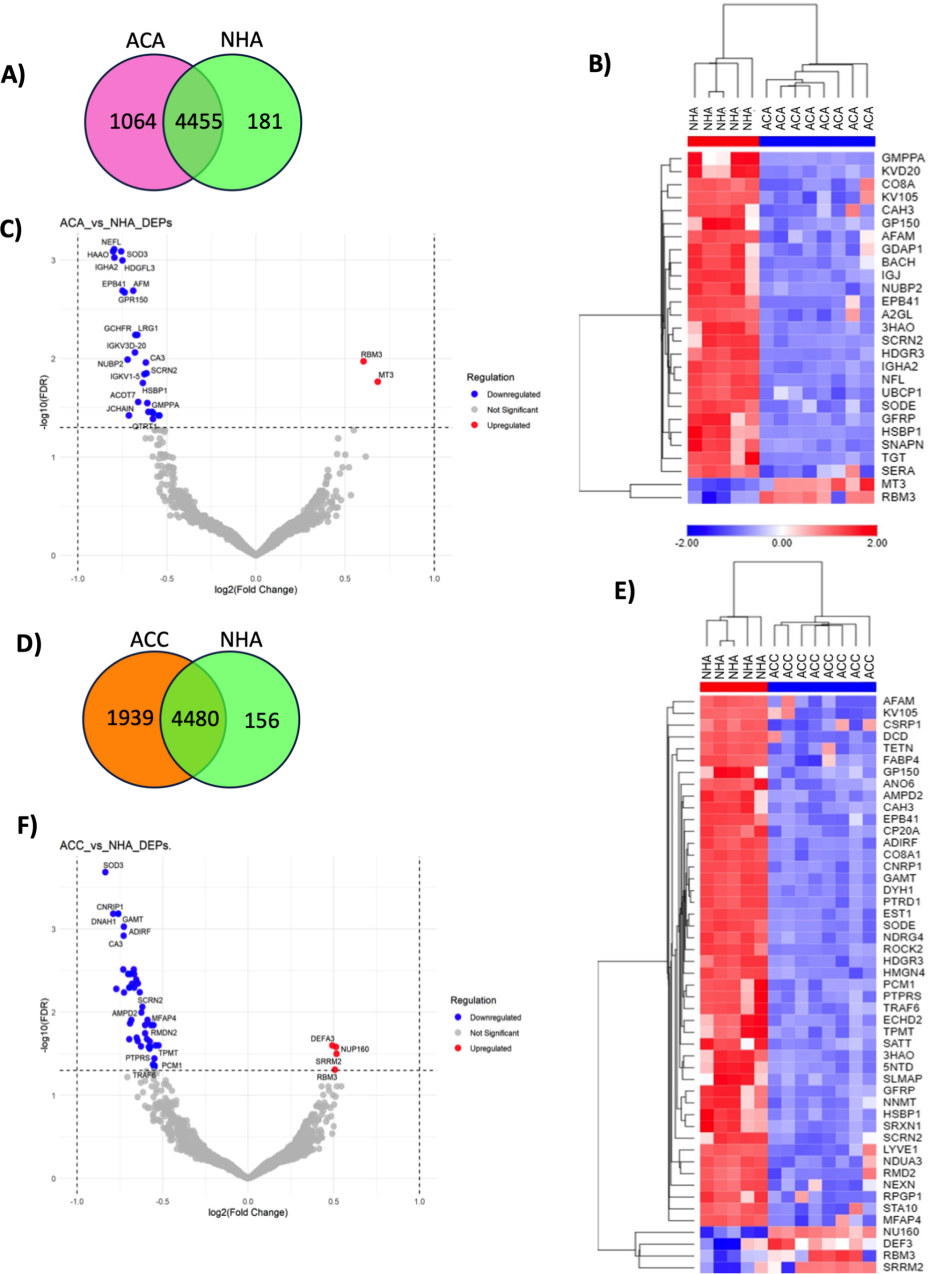
Differentially expressed protein of normal adrenal and adrenocortical tumor. (A) The total number of proteins expressed in ACA and NHA, the overlapping part of the circle indicates the number of proteins expressed in both groups; (B) heatmap of DEPs identified in ACA and NHA; (C) volcano plots for significantly differentially expressed proteins ACA vs NHA; (D) the number of total proteins expressed in ACC and NHA; (E) heatmap of DEPs identified in ACC and NHA; (F) volcano plots for significantly differentially expressed proteins ACC vs NHA. The -log10 (FDR) is plotted against log2 (Fold-Change); ACA = adrenocortical adenoma, NHA = normal human adrenal, ACC = adrenocortical carcinoma, DEPs = differentially expressed proteins, FDR = false discovery rate.

In Figure 4A, we compared PMAH and NHA, identifying 975 proteins in PMAH, 215 in NHA, and 4421 proteins expressed in both. There were 81 DEPs between PMAH and NHA, with 72 downregulated in PMAH, with AFM, CA3, IGKV1–5, ADIRF, and LRG1 as the top five downregulated proteins, and 9 upregulated proteins, with RBM3, RIGI, SPAG9, and SRRM2 proteins as the top four upregulated (Figure 4B,C). These protein expression analyses highlighted a distinct separation of the adrenal neoplasms studied (ACA, ACC, PMAH) and normal adrenal tissue, indicating that adrenocortical neoplasms exhibit distinct protein profiles compared to normal tissue.

**Figure 4:**
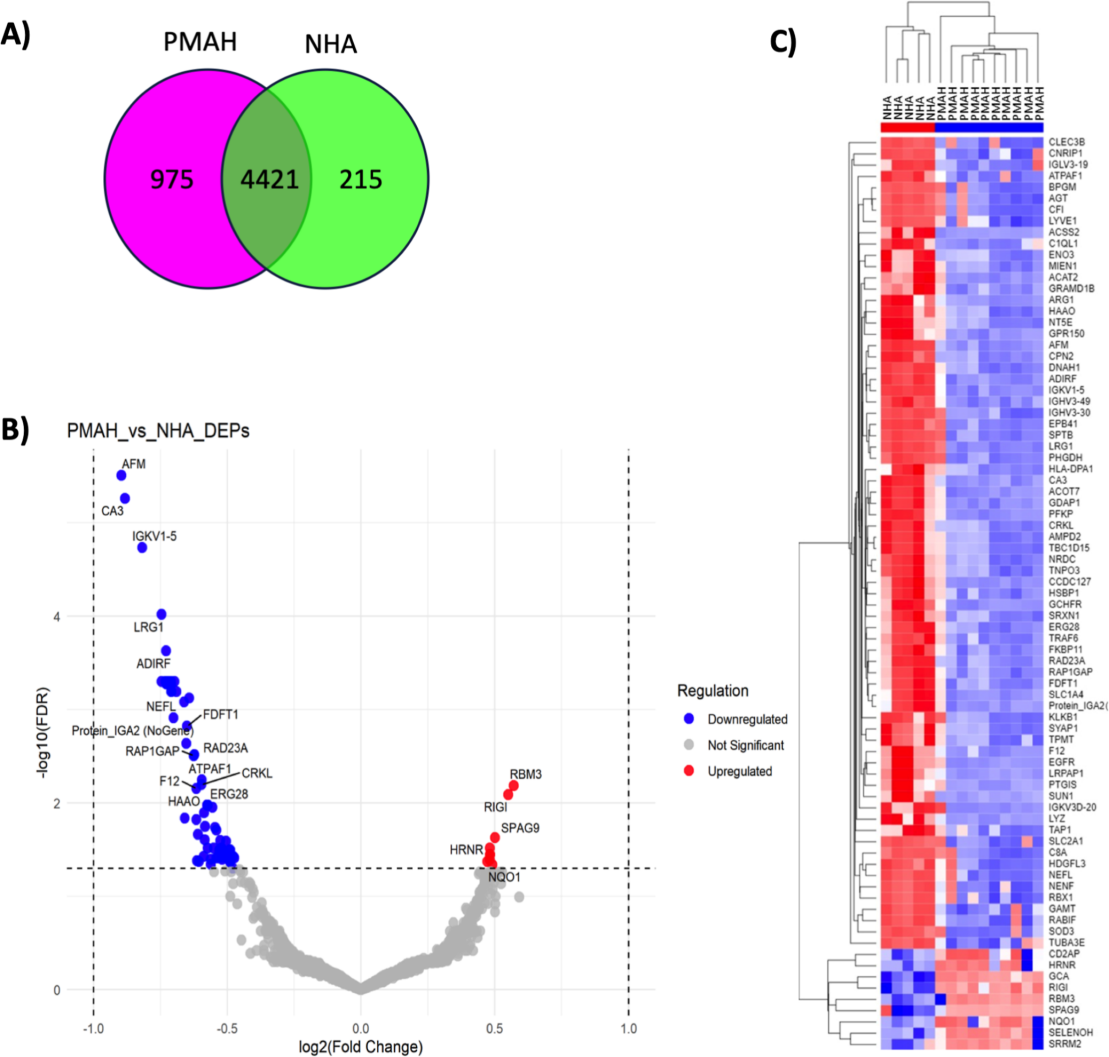
Differentially expressed protein of normal adrenal and hyperplasia. (A) The number of total proteins expressed in PMAH and NHA, the overlapping part of the circle indicates the number of proteins expressed in both groups; (B) heatmap of DEPs identified in PMAH and NHA; (C) volcano plots for significantly differentially expressed proteins PMAH vs NHA. The –log 10 (FDR) is plotted against log2 (Fold Change). PMAH = primary macronodular adrenal hyperplasia, NHA = normal human adrenal, DEPs = differentially expressed proteins, FDR = false discovery rate.

### Pairwise Comparisons of DEPs Between ACC/ACA and PMAHwt/PMAHw

Pairwise comparisons of DEPs between ACA and ACC (Figure 5) and between PMAHwt and PMAHw samples (Figure S3) were also conducted. In Figure 5A, 221 proteins were identified in ACA, 1111 in ACC, and 5308 were commonly observed in both tumor samples. The analysis of DEPs between ACC and ACA revealed the highest number of DEPs, with 64 upregulated in ACC and 48 downregulated (Figure 5 B,C). The top 10 DEPs upregulated in ACC included: PHGDH (SERA), PDCD4, MAGED2, ERAP2, FTO, DUSP23, UBLCP1, NSUN2, TP53BP1, and NUP107. The downregulated DEPs were RMDN2, CSRP1, ACAD11, PACSIN3, HAGH (GLO2), ALG5, NEXN, ABLIM1, LBP, and SYNPO2.

**Figure 5:**
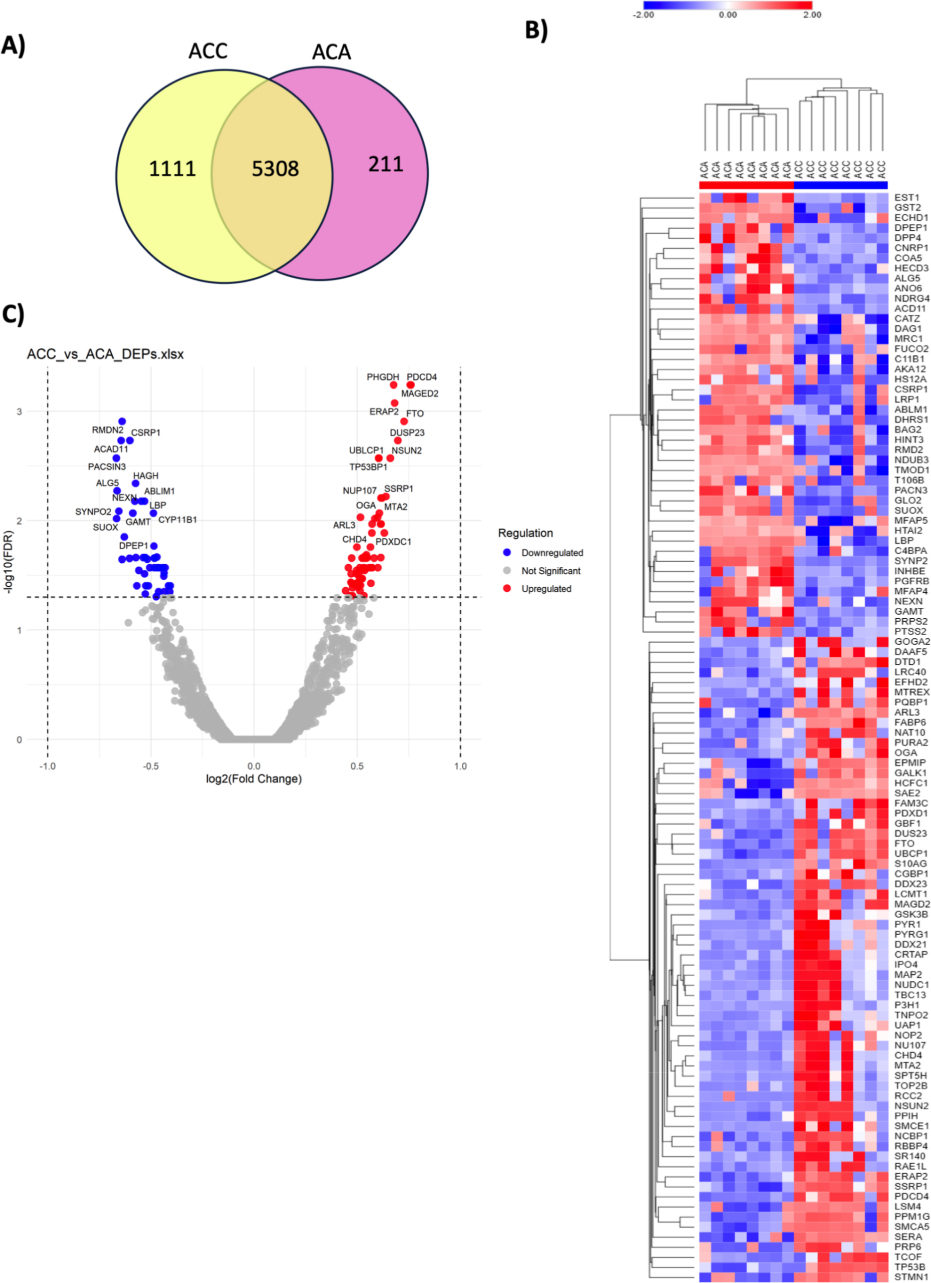
Differentially expressed protein of adrenocortical tumors. (A) The number of total proteins expressed in ACC and ACA, the overlapping part of the circle indicates the number of proteins expressed in both groups; (B) heatmap of DEPs identified in ACC and ACA; (C) volcano plots for significantly differentially expressed proteins ACC vs ACA. The log 10 (FDR = false discovery rate) is plotted against log 2 (Fold Change). ACC = adrenocortical carcinoma, ACA = adrenocortical adenoma, DEPs = differentially expressed proteins.

In Figure S3A, 717 proteins were identified in PMAHwt, 203 in PMAHw, and 4475 were commonly observed in both samples. The analysis of DEPs between PMAHwt and PMAHw showed the fewest DEPs, with 12 upregulated in PMAHw, including NOSTRIN, PCP4, C4BPB, and SERB (PSPH) as the top four upregulated proteins. Additionally, 4 proteins were downregulated in PMAHw: EST2 (CES2, TERT), PNPT1, RDH13, and STAB 1 (Figure S3B,C).

Network enrichment analysis using GO and STRING platforms of 64 ACC upregulated proteins relative to ACA identified biological processes with the highest signal, including pathways such as positive epigenetic regulation, regulation of *TP53* activity through acetylation, SUMOylation, and chromatin remodeling, as well as stem cell differentiation and others, with the lowest FDR (Figure S4).

### Reproducibility and Validation of Proteomic Data from ACC-USP Using an Independent ACC Cohort, TCGA, and Literature

A comparative analysis of proteomic profiles between studies conducted at the University of São Paulo (USP, *n* = 8) and Seoul National University Hospital (SNUH, *n* = 58) revealed significant quantitative agreement (Figure 6; Table S5). A total of 7350 proteins were identified in the ACC_USP proteome and 8302 in the ACC_SNUH study, with an overlap of 4201 proteins (Figure 6A). Keeping only proteins with valid values in at least 35% of samples from each group resulted in 3024 proteins for further analysis. The global average similarity between proteins was 99.8%, demonstrating remarkable quantitative agreement across proteomes. GSI analysis classified most proteins as having very high similarity, which was confirmed by Bland-Altman results showing negligible mean differences and no significant disparities (Z-score >1.96), indicating substantial similarity between groups (Figure 6B). Using an improved 4-fold cross-validation method, proteins were ranked based on a combined score that included both correlation and stability. The approach was tested on data subsets (ACC_SNUH × NHA_USP and ACC_SNUH × ACA_USP), confirming its robustness for multicenter applications. The validation of DEPs confirmed 37 (75.5%) of the 49 proteins identified as DEPs between ACC_SNUH and NHA_USP (Figure 6C), while 92 (82.1%) of the 112 proteins were identified as DEPs between ACC_SNUH and ACA_USP (Figure 7).

**Figure 6:**
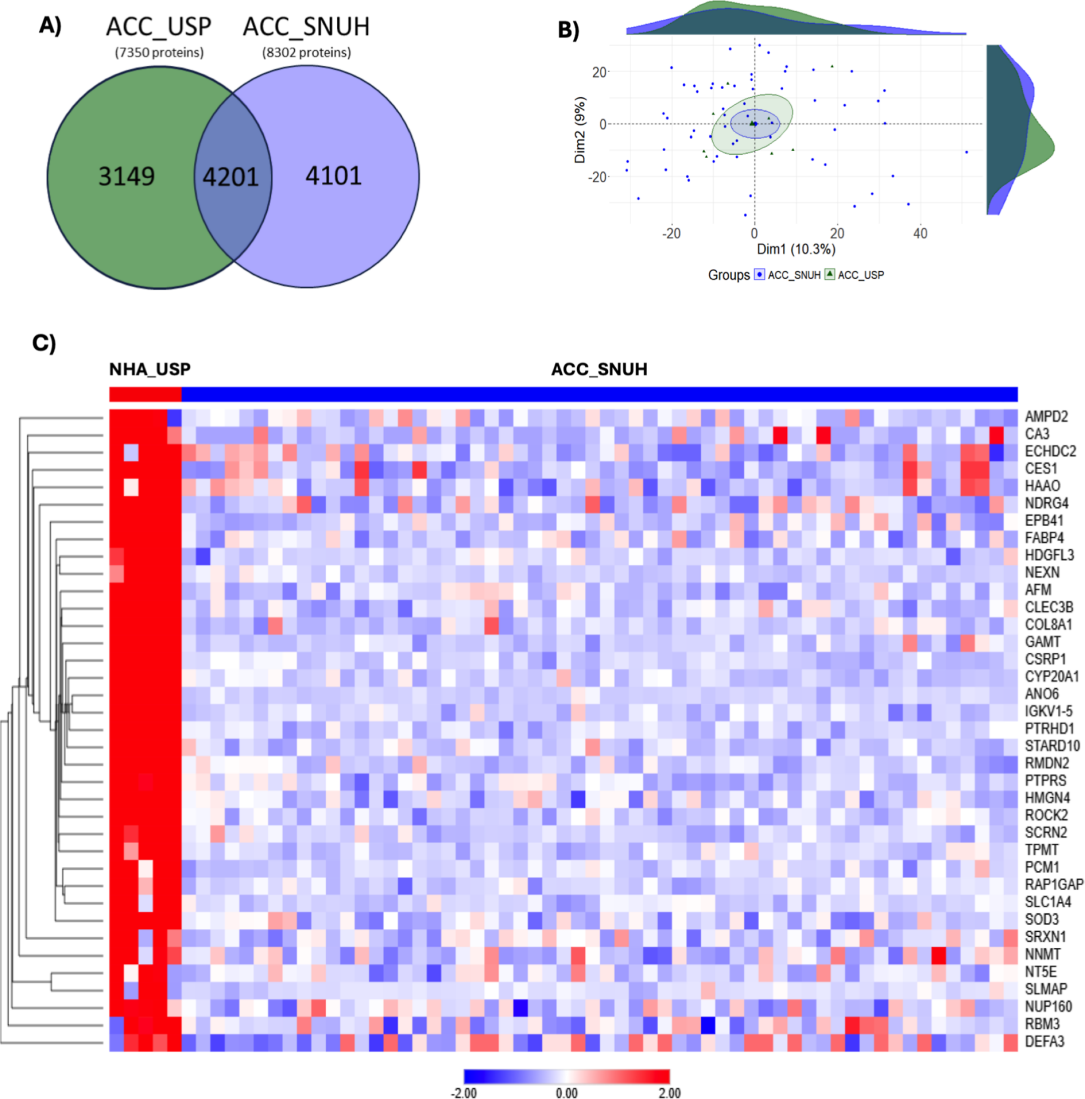
Cross-validation of proteomic signatures of neoplasia from USP and SNUH. (A) Proteomic data from adrenocortical carcinoma at the University of São Paulo (ACC_USP) versus Seoul National University Hospital (ACC_SNUH); (B) Principal Component Analysis (PCA) to show similarity among samples of ACC_USP (*n* = 8) and ACC_SNUH (*n* = 58); (C) Heatmap of normal human adrenal (NHA_USP) versus ACC_SNUH. NHA = normal human adrenal; ACC = adrenocortical carcinoma; DEP = differentially expressed proteins.

**Figure 7:**
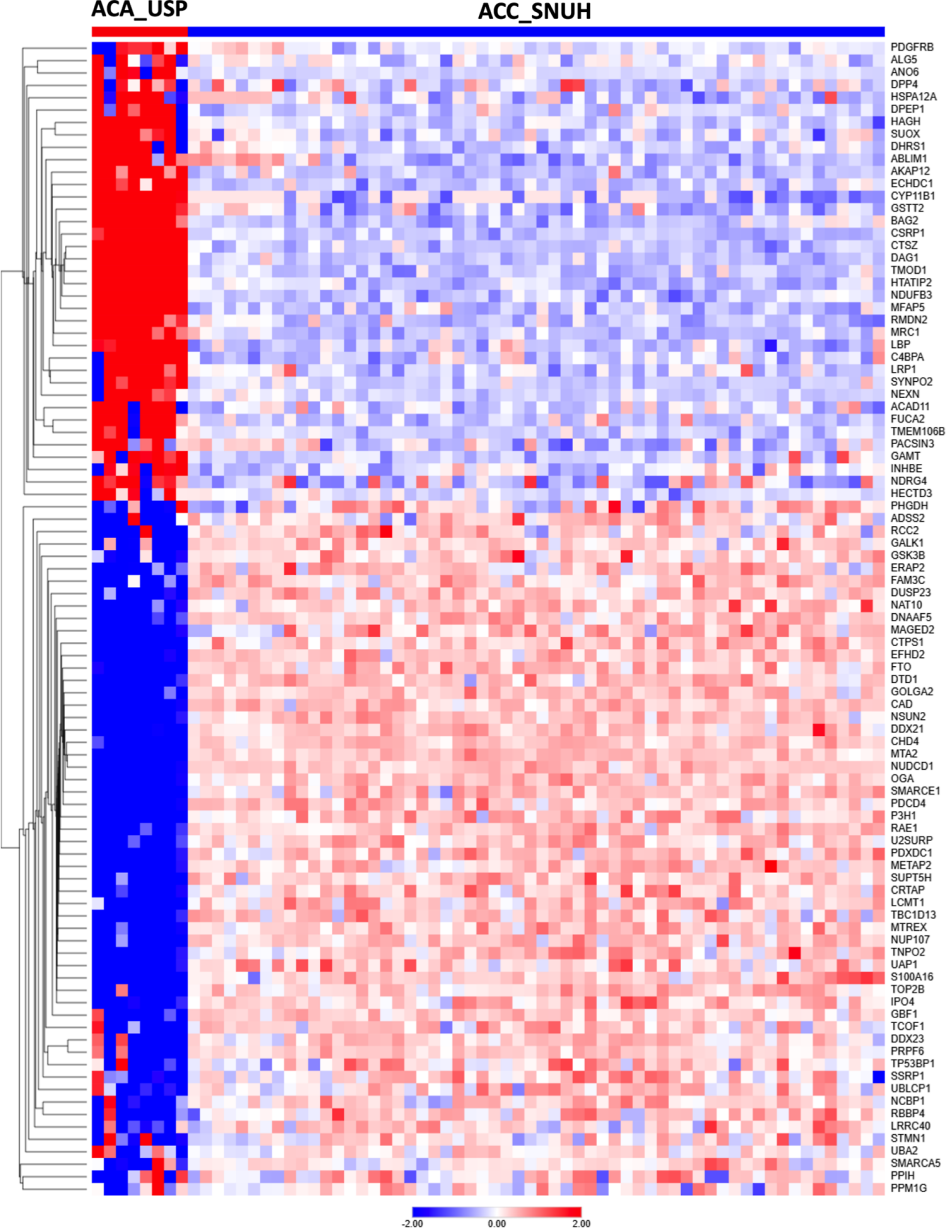
Cross-Validation of proteomic signatures of neoplasia from USP and SNUH. Heatmap of DEPs of adrenocortical adenoma of the University of São Paulo (ACA_USP, *n* = 8) versus adrenocortical carcinoma of Seoul National University Hospital (ACC_SNUH, *n* = 58). DEP = differentially expressed proteins.

Additionally, the use of TCGA identified five targets genes upregulated in ACC, Ubiquitin-like modifier activating enzyme 2 (*UBA2*), Golgi brefeldin A resistant guanine nucleotide exchange factor 1 (*GBF1*), glycogen synthase kinase 3 beta (*GSK3B*), DExD-box helicase 21­(*DDX21*) and EF-hand domain family member D2 (*EFHD2*) based on tumor staging, overall survival, and progression-free interval (Figure 8).

**Figure 8:**
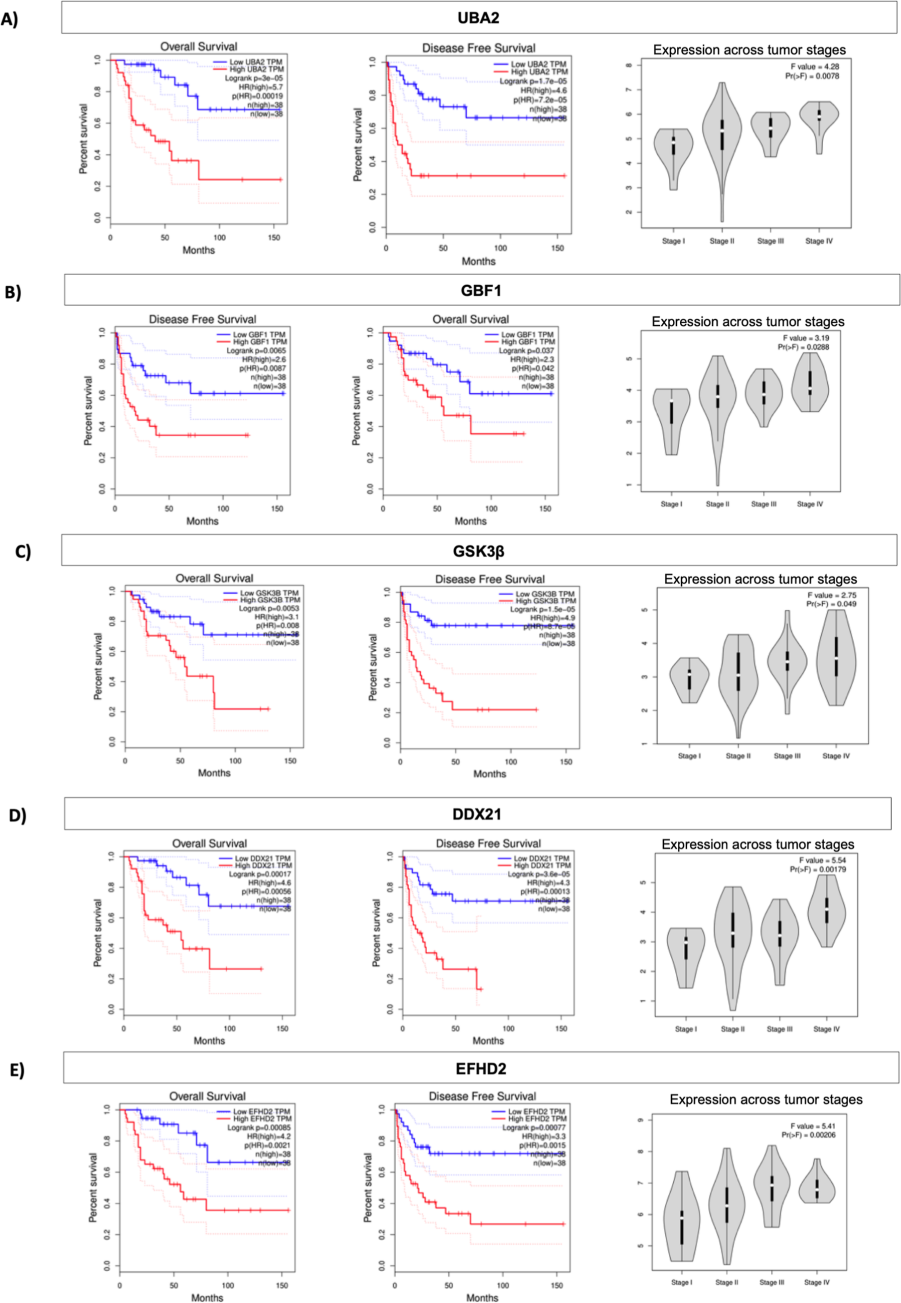
High expression of UBA2, GBF1, GSK3B, DDX21, and EFHD2 is associated with aggressive behavior and poor patient outcomes in ACC. Kaplan–Meier survival plot of overall survival (OS), disease-free survival (DFS), and expression across tumor stages of (A) Ubiquitin-like modifier activating enzyme 2 (UBA2); (B) Golgi brefeldin A resistant guanine nucleotide exchange factor 1 (GBF1), Glycogen synthase kinase 3 beta (GSK3B), DExD-box helicase 21­(DDX21) and EF-hand domain family member D2 (EFHD2) expression from TCGA analysis. *n* = 38.

These proteins of these genes were found to be upregulated in ACC according to our proteomic data and validated in the SNUH cohort, and are associated with aggressive behavior and poor patient outcomes in ACC. Furthermore, biomarkers reported in the literature in ACC, such as Stathmin 1 (*STMN1*),^20,21^ N-myc downregulated gene family 4 (*NDRG4*),^22,23^ and phosphoglycerate dehydrogenase (*PHGDH*),^24^ validated in our proteomic analysis, were also associated with aggressive behavior and poor patient outcomes in ACC (Figure S5).

## Discussion

LC–MS/MS has emerged as a valuable tool for enhancing our understanding of biology and diagnosing various neoplasms. Few proteomic studies have been conducted on adrenal tumors.^19,25–27^ To our knowledge, two studies, Yang et al.^25^ and Jang et al.,^19^ have utilized LC–MS/MS analysis to explore the proteome profile of adrenal tumors. However, these studies were limited to comparing tumor samples. Furthermore, they did not expand their analyses to include comparisons with normal adrenal tissue or other alterations of the adrenal cortex, such as PMAH. In this exploratory study, we compared the proteomes of normal adrenal tissue, ACA, ACC, and PMAH, identifying 7350 proteins that could help understand the biology of the adrenal cortex in both health and disease.

### Identification of DEPs by Comparing Neoplasms with NHA

The identification of DEPs comparing ACA, ACC, and PMAH with NHA samples revealed the downregulation of RNA-binding motif protein 3 (RBM3) in all neoplasms analyzed when compared to NHA.

RBM3 is a member of the highly conserved RNA-binding proteins that participate in cellular processes by binding to the mRNA of target genes.^28^ Under normal physiological conditions, RBM3 is essential for cell survival and proliferation; however, it is dysregulated in various cancers. Its expression is reduced, for instance, in urothelial bladder cancer^29^ and nonsmall cell lung cancer^30^ but increased in epithelial ovarian cancer^31^ and prostate cancer.^32^ In both instances where RBM3 is upregulated, this correlates with a favorable prognosis. The dysregulation of RBM3 suggests that it may be involved in tumor progression,^33^ particularly in adrenocortical neoplasms, as its negative expression has been observed in ACA, ACC, and PMAH. Conversely, superoxide dismutase 3 (SOD3) was found to be downregulated in both ACA and ACC. SOD3 is a metalloenzyme primarily located in the extracellular space, either in a soluble form or associated with the ECM. This enzyme catalyzes the conversion of superoxide radicals into hydrogen peroxide and oxygen, potentially protecting tissues from oxidative stress; however, it is downregulated in ACA and ACC compared to normal adrenal tissue. SOD3 expression is notably higher in the lungs, kidneys, uterus, placenta, and cardiovascular system, and oncogenesis generally leads to the downregulation of SOD3.^34^ However, its precise role remains uncertain.^35^ The reduced levels of SOD3 in tumors result from epigenetic silencing, mRNA targeting by oncomiR-21, mutations in the SOD3 gene, and alterations in the ECM’s structure, composition, and dynamics.^36^ For example, SOD3 inhibits the activity of metalloproteinases and the expression of heparanase, an ECM-degrading enzyme that promotes cancer growth, metastasis, and angiogenesis by facilitating the degradation of heparan sulfate.^37^ In contrast, SOD3 levels exhibit a positive correlation with the growth of certain benign tumors associated with sustained activation of the MAPK pathway, either through direct regulation of tyrosine kinase receptor phosphorylation or small Rho/Ras GTPase signaling.^35^

Aside from RBM3 and SOD3, no other proteins are positively or negatively regulated when comparing ACA and PMAH with NHA. However, the DEP profiles of ACC and PMAH exhibit similarities, including the downregulation of CA3 and ADIRF, and the upregulation of SRRM2.

Carbonic anhydrase 3 (CA3) and adipogenic regulatory factor (ADIRF) are downregulated in ACC and PMAH, whereas serine/arginine repetitive matrix 2 (SRRM2) is upregulated in both. The CA3, thioredoxin, and glutathione redox systems are key cellular regulators of redox and pH balance. CA3 has been shown to bind to other proteins, which can modulate autophagy and proinflammatory signaling in cancer cells. CA3 has also been associated with epithelial–mesenchymal transition processes, which promote cancer cell metastasis. Conversely, CA3 overexpression activates the PI3K/AKT/mTOR pathway, leading to upregulated cell growth and inhibited autophagy. It is unknown whether CA3 modulates cancer progression through its reported antioxidant functions.^38^

ADIRF is expressed in various tissues, and its function extends beyond regulating apoptosis and cell growth. ADIRF expression is altered in several malignancies, yet its role remains contradictory. The absence of ADIRF in prostate cancers is associated with lymph node metastasis and poorer survival outcomes, suggesting that ADIRF may act as a cancer suppressor gene.^39^ In ovarian cancer, ADIRF is associated with a poor prognosis, and its overexpression induces various miRNAs involved in multiple signaling pathways, including the Wnt, TGF-β, MAPK, and Jak-STAT pathways.^40^ Also, in colorectal cancer, high ADIRF expression potentially facilitates cisplatin resistance and poor outcomes in cancer patients.^41^ SRRM2 is an unstructured serine/arginine-rich (SR) protein that serves as a spliceosomal component and one of the core scaffold proteins necessary for properly forming nuclear speckles, where mRNA maturation and splicing may occur.^42^ Recently, Kellner et al.^43^ demonstrated that SRRM2 appears on the surface of cancer cells, identifying it as a promising new target molecule for targeted cancer therapy.

### Identification of Upregulated DEPs in ACAs and ACCs Compared with NHA

Regarding the upregulated DEPs identified only in ACAs and ACCs about NHA, metallothionein 3 (MT3) was identified in ACAs; in ACCs, the upregulated DEPs were DEFA3 and NUP160.

MT3 is abundant in the brain but appears to be ubiquitously distributed in various tissues. The most essential function of MT3 is its antioxidant action, which occurs through the oxidation–reduction reaction under oxidative stress conditions, thereby preventing damage to cellular organelles. This oxidation–reduction reaction is critical in suppressing the early stages of diseases such as stroke, cardiomyopathy, diabetic retinopathy, and cancer. Additionally, MT3 regulates autophagy and contributes to alterations in cell metabolism and cellular signaling pathways.^44^ Interestingly, MT3 mRNA level was higher in the aldosterone-producing adenoma than in the cortisol-producing adenoma. MT3 immunoreactivity was detected in the zona glomerulosa of normal adrenal, suggesting a role in aldosterone-producing adrenocortical cells^44^ Therefore, these findings, along with ours, indicate that the role of MT3 warrants further investigation in ACA and normal adrenal tissue.

Defensins are a vital component of the innate immune system, safeguarding against microbial infections, modulating immune responses, and enabling tissue development and regeneration.^45^ Recent studies have underscored the intricate interplay between defensins and cell cycle regulation.^46^ Defensin alpha 3 (DEFA3) has been demonstrated to influence the cell cycle by acting as an oncogene in 31 cancer types, including ACC.^47^ Reduced DEFA 1–3 expression may shield pleomorphic adenomas from malignant transformation into adenocarcinomas.^48^ Overall, this and our evidence position DEFA3 as a key focus for future research in ACC.

NUP160 is the one constituent nucleoporin that forms part of NUP107–160, the largest nuclear pore complex (NPC) subunit. The NPC facilitates the movement of molecules in both directions between the cell nucleus and cytoplasm, with NUP107–160 being a critical subcomplex necessary for multiple functions, such as NPC assembly during interphase and the segregation of chromosomes during mitosis. Knockdown of NUP160 inhibits cell proliferation, induces apoptosis, promotes autophagy and cell migration, and alters the expression and localization of podocyte-related molecules in mouse podocytes.^49^ In patients with chronic myeloid leukemia (CML), the expression of NUP160 is significantly elevated in peripheral blood or cell lines, decreasing the sensitivity of CML to imatinib.^50^ Conversely, in human angiosarcoma, the gene fusion NUP160-SLC43A3 causes truncation of NUP160, thereby enhancing cell proliferation.^51^

### Identification of Relevant DEPs in ACC Compared to ACA

Considering the most significant top ten DEPs between ACCs and ACAs, along with the previous description of their presence in adrenocortical tissue tumors, we selected phosphoglycerate dehydrogenase (PHGDH) and fat mass and obesity-associated protein (FTO) upregulated in ACC and cysteine and glycine-rich protein 1 (CSRP1) and actin-binding LIM protein 1 (ABLIM1) downregulated in ACC.

According to our findings, PHGDH expression was higher in ACC than in ACA. Kim et al.^24^ showed that PHGDH was associated with shorter overall survival and, together with glucose transporter 1 (GLUT1), correlates with poor prognosis in adrenal tumors. Notably, the inhibition of PHGDH limits tumor growth.^52^

FTO downregulates the N6-methyladenosine (m6A) level, and many studies have suggested that the m6A modification plays a vital role in various malignancies. FTO is elevated in cervical squamous cell carcinoma compared to normal tissues, enhancing chemo-radiotherapy resistance by regulating β-catenin expression through reduced m6A levels. In contrast with the upregulation of FTO in ACC compared with ACA identified here, FTO was found to be downregulated in an observational study of m6A-related expression in ACC tissues.^53^ Given the contrast between the results and the aberrantly activated Wnt/β-catenin pathway, which is implicated in adrenocortical carcinogenesis,^54^ further studies on the role of FTO in ACCs should be pursued.

Xu et al.^55^ identified downregulated hub genes in ACC, one of which was CSRP1, consistent with our findings in the proteomic analysis of ACCs concerning ACA. CSRP1 is hypothesized to be a tumor suppressor gene in colorectal cancer^56^ and is inactivated in liver cancer due to abnormal methylation.^57^ This suggests that CSRP1 may be a tumor suppressor gene in ACC, warranting further investigation.

In agreement with our analysis’s identification of downregulation of the ABLIM1 protein in ACC, a microarray analysis of ACCs and ACAs also identified ABLIM1, which was validated by qPCR, as significantly downregulated in ACCs compared to ACAs.^58^ The localization of *ABLIM1* to 10q25, a region frequently deleted in human cancers that contains several tumor suppressor genes, identified it as a candidate for a tumor suppressor in melanomas.^59^ Kwong & Chin^60^ proved that knockdown of the Ablim1 gene using RNAi enhances invasion in vitro. In esophageal cancer (EC), ABLIM1 could be used as a diagnostic biomarker for EC.^61^ These findings suggest that ABLIM1 may also be a tumor suppressor in ACC.

### Identification of Relevant DEPs in PMAHwt Compared to PMAHw

Considering the four most significant DEPs between PMAHwt and PMAHw, along with literature data, we selected Purkinje cell protein 4 (PCP4), which is upregulated in PMAHw, and telomerase reverse transcriptase (TERT), which is downregulated in PMAHw. PCP4 is a calmodulin-binding protein that enhances the association and dissociation of calcium with calmodulin. It has been previously detected in aldosterone-producing adenomas.^62,63^ The upregulation of PCP4 in aldosterone-producing human adenomas promotes growth via the AKT and AMPK pathways, and PCP4 mRNA expression level was positively correlated with tumor size in APAs.^64^ Interestingly, adrenals with PMAHw were significantly larger and had more nodules than those with *ARMC5* wild-type presentations,^2,65^ suggesting that PCP4 may be related to nodule size in PMAH and to the *ARMC5* mutation.

In contrast with PCP4, ribonucleoprotein polymerase TERT, which maintains telomere ends, is downregulated in PMAHw. It is typically repressed in postnatal somatic cells, leading to the progressive shortening of telomeres and cellular senescence. In one case description of unilateral adrenal cortical hyperplasia, telomerase activity was not detected in either the nodule or nonhyperplastic adrenal cortical tissue.^66^ Kanauchi et al.,^67^ described that hTERT expression was increased in ACC compared with normal tissues, ACA, and hyperplasia. However, the difference in TERT expression between PMAHw and PMAHwt is intriguing and warrants further exploration.

### Reproducibility and Validation of Proteomic Data from ACC-USP Using an Independent ACC Cohort, TCGA, and Literature

Cross-validation of the proteomic data set using the DEPs identified by Jang et al.,^19^ who used LC–MS/MS, formalin-fixed, paraffin-embedded tumor samples, helps strengthen our findings from the proteomic analysis of ACC. Through the application of machine learning algorithms and survival analysis, they identified a panel of prognostic protein biomarkers, HNRNPA1, LTBP4, MRPS23, POLDIP2, and WBSCR16. Notably, we validated four of these five prognostic proteins, HNRNPA1, LTBP4, MRPS23, and POLDIP2. This cross-validation confirms the reliability of our data and supports the use of these proteins as predictive biomarkers, strengthening the existing proteomic signature. The cross-analysis of the data enabled the confirmation of 75,5% of the DEPs identified between NHA and ACC, and 82,1% of the DEPs between ACC and ACA, providing a solid foundation for subsequent cross-validation analyses at the mRNA level using data from TCGA.

One of the targets selected by TGCA and validated in our proteomic analysis was the protein UBA2. Previous reports have demonstrated the role of UBA2 in various cancer types, whose expression is positively correlated with the tumor promoter EZH2.^68^ SAE1 and UBA2 form a heterodimer that functions as a SUMO-activating enzyme for the SUMOylation of proteins.^69^ Interestingly, analysis of the TCGA in ACC reveals that UBA2 expression is positively correlated with SAE1, SUMO1, UBE2I (also known as UBC9), and TRIP6. Proteins involved in the SUMO pathways play a crucial role in human tumorigenesis, and SUMO proteins may be utilized to monitor various human tumors;^70^ therefore, further investigation is still needed to validate SUMO alterations as biomarkers for ACC.

While this study provides valuable proteomic insights, several limitations should be acknowledged, including a small sample size of normal and neoplastic tissues. Obtaining human adrenal gland samples is challenging due to ethical and practical constraints. Technical limitations include potential interference from cellular remnants during proteomic analysis and the inability to reverse enzymatically induced or spontaneously occurring protein cross-links, which may affect protein identification. Although LC–MS/MS offers broad proteome coverage, detecting low-abundance proteins remains challenging without the use of specific enrichment strategies. Finally, the biological complexity of adrenal neoplasms underscores the need for integrated multiomics approaches that combine genomic, transcriptomic, and functional analyses to comprehensively understand the biology of tissue neoplasm alterations.

## Conclusion

This study presents one of the most comprehensive proteomic analyses to date of the normal human adrenal cortex and adrenocortical neoplasms (ACA, ACC, and PMAH), resulting in a high-quality proteomic data set that represents a valuable resource in the field. This data set enabled robust comparative analyses and the identification of potential biomarkers and therapeutic targets, while also serving as a reference framework for future studies.

## Supplementary Material













## Notes

Figure S1. The histopathological characteristics of (A) normal human adrenal (NHA); (B) adenoma adrenocortical (ACA); (C) carcinoma adrenocortical; (D) Primary macronodular hyperplasia adrenal (with mutation in the *ARMC5* gene (PMAHw)); (E) Primary macronodular hyperplasia adrenal (without mutation in the *ARMC5* gene (PMAHwt). Figure S2. Correlation of proteomics-identified targets with clinical data in ACC using Gene Expression Profiling Interactive Analysis (GEPIA platform); Overall Survival (OS); disease-free survival (DFS). *SRRM2* = serine/arginine repetitive matrix 2; *NUP160* = nuclear pore complex protein; *RBM3* = RNA-binding motif protein 3. Figure S3. Differentially expressed protein of hyperplasia with and without *ARMC5* mutation. (A) The number of total proteins expressed in PMAHwt and PMAHw, the overlapping part of the circle indicates the number of proteins expressed in both groups; (B) Heatmap of DEPs identified in PMAHw and PMAHwt; (C) Volcano plots for significantly differentially expressed proteins PMAHwt vs PMAHw. The –log 10 (FDR) is plotted against log 2 (Fold-Change). PMAHw = primary macronodular adrenal hyperplasia with *ARMC5* mutation, PMAHwt = primary macronodular adrenal hyperplasia without *ARMC5* mutation, DEPs = differentially expressed proteins, FDR = false discovery rate. Figure S4. Biological processes identified by network enrichment analysis using Gene Ontology (GO) and STRING platforms of 64 adrenocortical carcinomas (ACC) upregulated proteins relative to adrenocortical adenomas (ACA). FDR = false discovery rate. Figure S5. High expression of *STMN1* and *PHGDH* is associated with aggressive behavior and poor patient outcomes in ACC, whereas NDRG4 expression is the opposite. Kaplan–Meier survival plot of overall survival (OS), disease-free survival (DFS), and expression across tumor stages of (A) Stathmin 1 (*STMN1*); (B) phosphoglycerate dehydrogenase (*PHGDH*); (C) N-myc downregulated gene family 4 (*NDRG4*) expression from TCGA analysis. *n* = 38 (PDF)
Table S1: Total proteins identified in primary macronodular adrenocortical hyperplasia without *ARMC5* mutation (XLSX)
Table S2: Total proteins quantified in all analyzed samples (XLSX)
Table S3: Proteins identified specifically in each group and Proteins exclusively identified comparing all groups (XLSX)
Table S4: Proteins identified as up- or downregulated between the two conditions (XLSX)
Table S5: Cross-validation of DEPs in ACC-SNUH identified in ACC-USP on comparison between the two conditions (XLSX)

## Abbreviations

LC-MS/MS: Liquid chromatography-tandem mass spectrometry
NHA: normal human adrenal
ACA: adrenocortical adenomas
ACC: adrenocortical carcinomas
PMAH: primary macronodular adrenocortical hyperplasia
PMAHw with and PMAHwt without: *ARMC5* pathogenic variants
DEPs: Differentially expressed proteins.
