# Supplementary material for: Comparative Proteomic Profiling of Adrenocortical Neoplasia Using Mass Spectrometry
Source: J Proteome Res. 2025 Nov 21;24(12):6186–202. doi: 10.1021/acs.jproteome.5c00740 (PMC12687312; doi:10.1021/acs.jproteome.5c00740)
Supplement: Supplementary file 1 [file pr5c00740_si_001.pdf]

Cover/Title page

Supporting Information

Comparative Proteomic Profiling of Adrenocortical Neoplasia Using Mass Spectrometry

Jean Lucas Kremer[1], Henrique Sanchez Ortega[1], Talita Souza-Siqueira[2], Claudia Blanes Angeli[3], Leo Kei Iwai[4], Claudimara Ferini Pacicco Lotfi[1*]

1. Institute of Biomedical Sciences, Department of Anatomy, University of São Paulo, São Paulo, Brazil, Av. Prof. Lineu Prestes, 2415, Butantan, São Paulo, SP, 05508-000.

2. School of Medicine, Department of Clinical Medicine, University of São Paulo, Brazil, Av. Dr. Arnaldo, 455, Cerqueira César, São Paulo, SP, 01246903.

3. Institute of Biomedical Sciences, Department of Parasitology, University of São Paulo, Brazil, Av. Prof. Lineu Prestes, 1374, Butantan, São Paulo, SP, 05508-000.

4. Butantan Institute, Laboratory of Applied Toxicology, Center of Toxins, Immune-response and Cell Signaling LETA/CeTICS Laboratory, Brazil, Av. Vital Brasil, 1500 - Butantã, São Paulo, SP, 05503-900.

*Corresponding author: clotfi@usp.br.

Table of Contents

Figure S1. The histopathological characteristics of A) normal human adrenal (NHA); B) adenoma adrenocortical (ACA); C) carcinoma adrenocortical; D) Primary macronodular hyperplasia adrenal (with mutation in the ARMC5 gene (PMAHw); E) Primary macronodular hyperplasia adrenal (without mutation in the ARMC5 gene (PMAHwt). Supplementary_File_Figure S1.pdf.

Figure S2. Correlation of proteomics-identified targets with clinical data in ACC using Gene Expression Profiling Interactive Analysis (GEPIA platform); Overall Survival (OS); disease-free survival (DFS). SRRM2 = serine/arginine repetitive matrix 2; NUP160 = nuclear pore complex protein; RBM3 = RNA-binding motif protein 3. Supplementary_File_Figure S2.pdf.

Figure S3. Differentially expressed protein of hyperplasia with and without ARMC5 mutation. A) The number of total proteins expressed in PMAHwt and PMAHw, the overlapping part of the circle indicates the number of proteins expressed in both groups; B) Heatmap of DEPs identified in PMAHw and PMAHwt; C) Volcano plots for significantly differentially expressed proteins PMAHwt vs. PMAHw. The -log10 (FDR) is plotted against log2 (Fold-Change). PMAHw = primary macronodular adrenal hyperplasia with ARMC5 mutation, PMAHwt = primary macronodular adrenal hyperplasia without ARMC5 mutation, DEPs = differentially expressed proteins, FDR = false discovery rate. Supplementary_File_Figure S3.pdf.

Figure S4. Biological processes identified by network enrichment analysis using Gene Ontology (GO) and STRING platforms of 64 adrenocortical carcinomas (ACC) upregulated proteins relative to adrenocortical adenomas (ACA). FDR = false discovery rate. Supplementary_File_Figure S4.pdf.

Figure S5. High expression of STMN1 and PHGDH is associated with aggressive behavior and poor patient outcomes in ACC, whereas NDRG4 expression is the opposite. Kaplan–Meier survival plot of overall survival (OS), disease-free survival (DFS), and expression across tumor stages of A) Stathmin 1 (STMN1); B) phosphoglycerate dehydrogenase (PHGDH); C) N-myc downregulated gene family 4 (NDRG4) expression from TCGA analysis. n = 38. Supplementary_File_Figure S5.pdf.

Table S1 – Total proteins identified in primary macronodular adrenocortical hyperplasia without ARMC5 mutation. Supplementary_File_Table S1.xlsx.

Table S2 - Total proteins quantified in all analyzed samples. Supplementary_File_Table S2.xlsx.

Table S3 - Proteins identified specifically in each group and Proteins exclusively identified comparing all groups. Supplementary_File_Table S3.xlsx.

Table S4 - Proteins identified as up- or downregulated between the two conditions. Supplementary_File_Table S4.xlsx.

Table S5 - Cross-validation of DEPs in ACC-SNUH identified in ACC-USP on comparison between the two conditions. Supplementary_File_Table S5.xlsx.

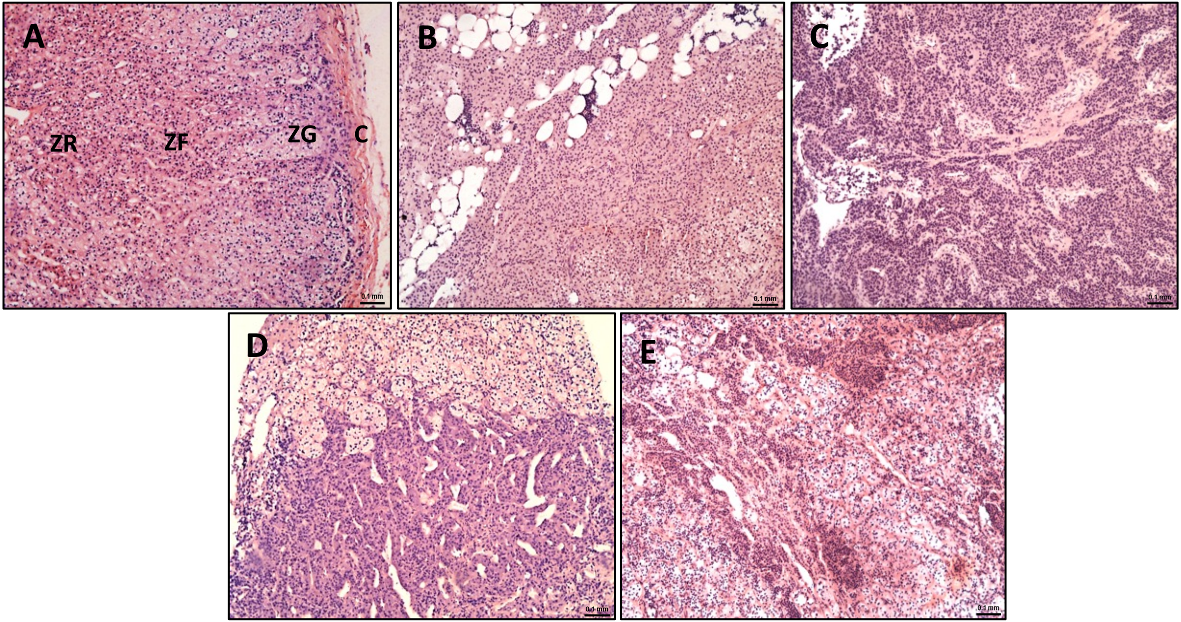

Figure S1. The histopathological characteristics of A) normal human adrenal (NHA); B) adenoma adrenocortical (ACA); C) carcinoma adrenocortical; D) Primary macronodular hyperplasia adrenal (with mutation in the ARMC5 gene (PMAHw); E) Primary macronodular hyperplasia adrenal (without mutation in the ARMC5 gene (PMAHwt). C = capsule; ZG = zone glomerulosa; ZF = zone fasciculada; ZR = zone reticular.

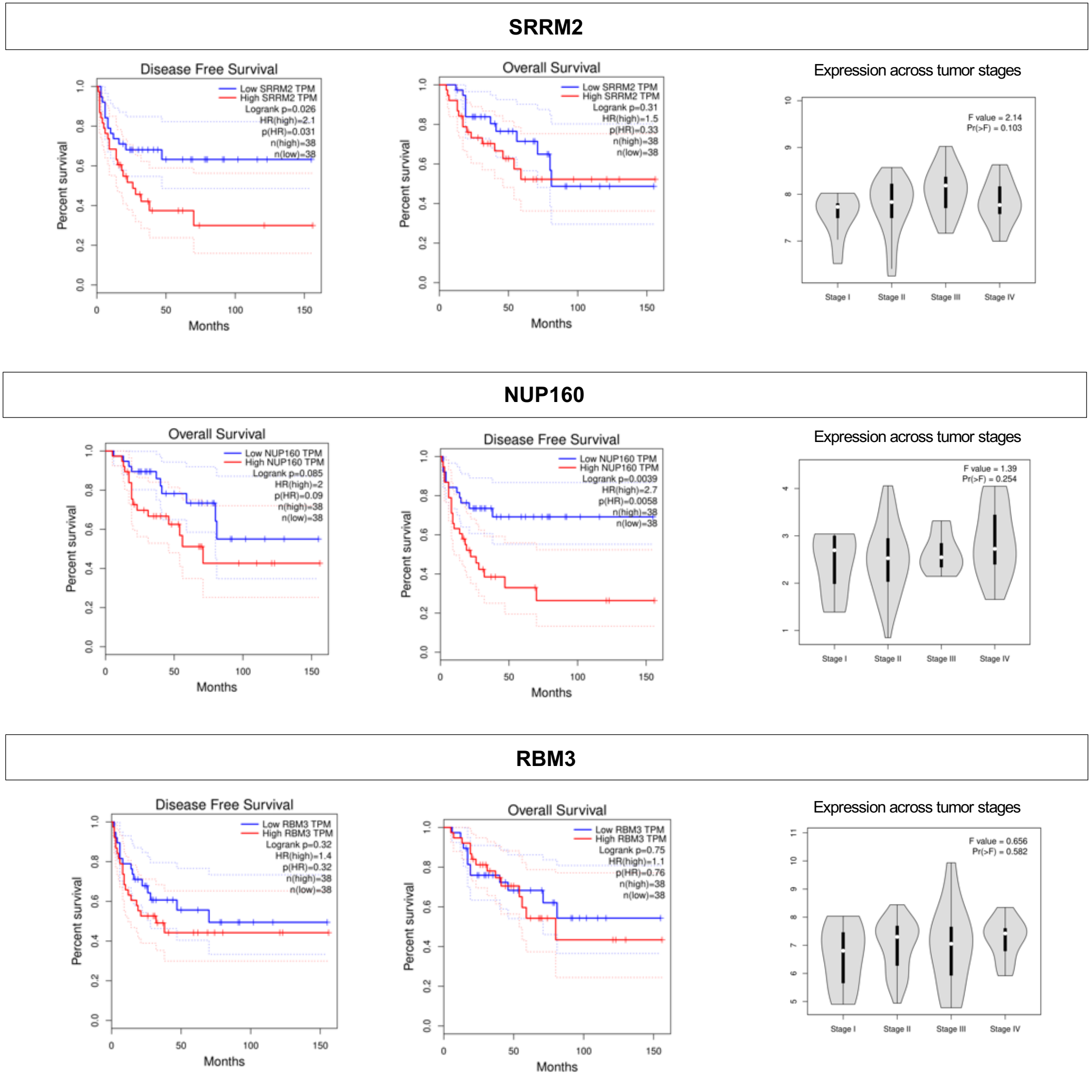

Figure S2. **Correlation of proteomics-identified targets with clinical data in ACC using Gene Expression Profiling Interactive Analysis (GEPIA platform)**; Overall Survival (OS); disease-free survival (DFS). SRRM2 = serine/arginine repetitive matrix 2; NUP160 = nuclear pore complex protein; RBM3 = RNA binding motif protein 3.

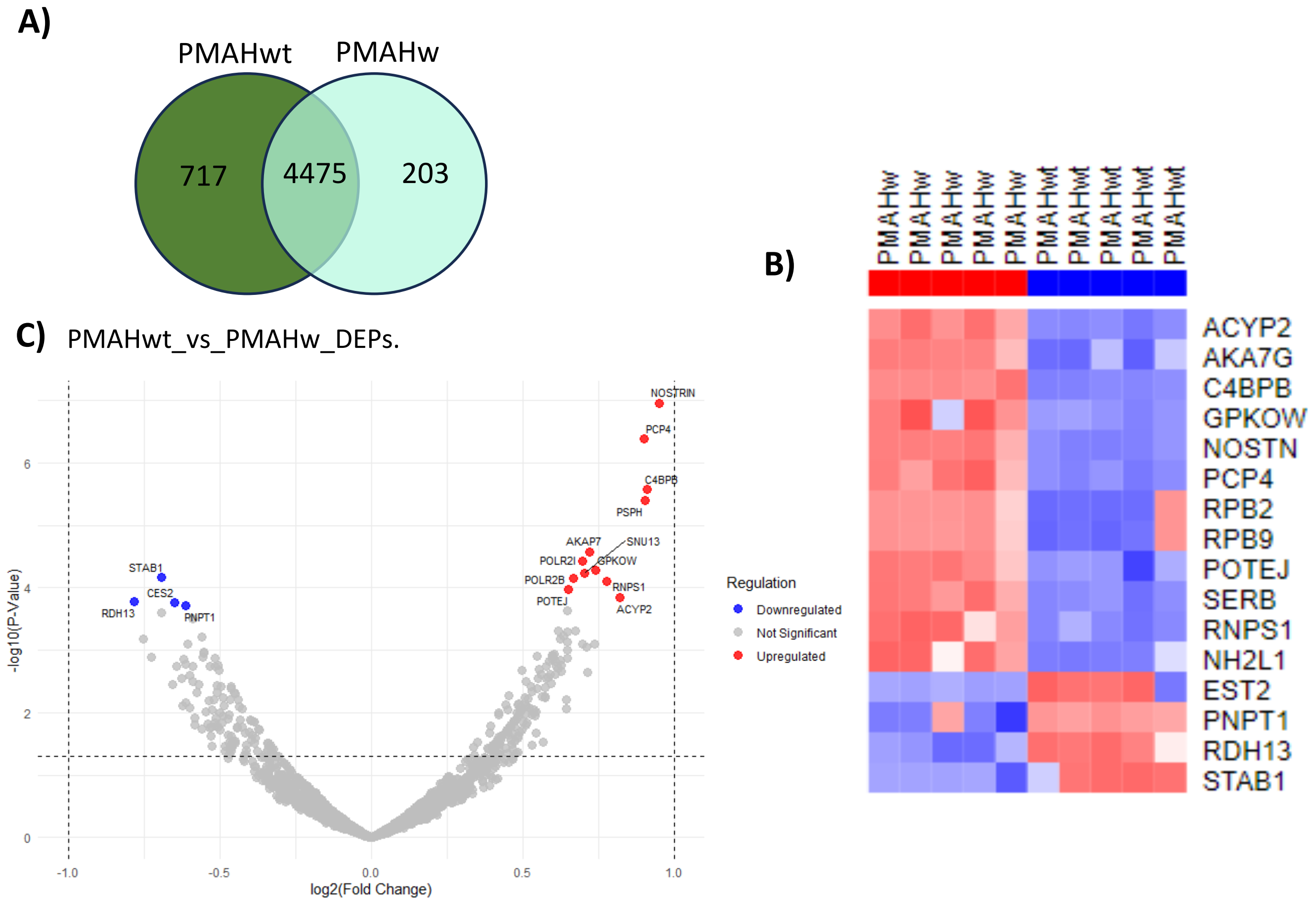

Figure S3. **Differentially expressed protein of hyperplasia with and without ARMC5 mutation.** A) The number of total proteins expressed in PMAHw and PMAHwt, the overlapping part of the circle indicates the number of proteins expressed in both groups; B) Heatmap of DEPs identified in PMAHw and PMAHwt; C) Volcano plots for significantly differentially expressed proteins PMAHw vs. PMAHwt. The -log10 (FDR) is plotted against log2 (Fold-Change). PMAHw = primary macronodular adrenal hyperplasia with ARMC5 mutation, PMAHwt = primary macronodular adrenal hyperplasia without ARMC5 mutation, DEPs = differentially expressed proteins, FDR = false discovery rate.

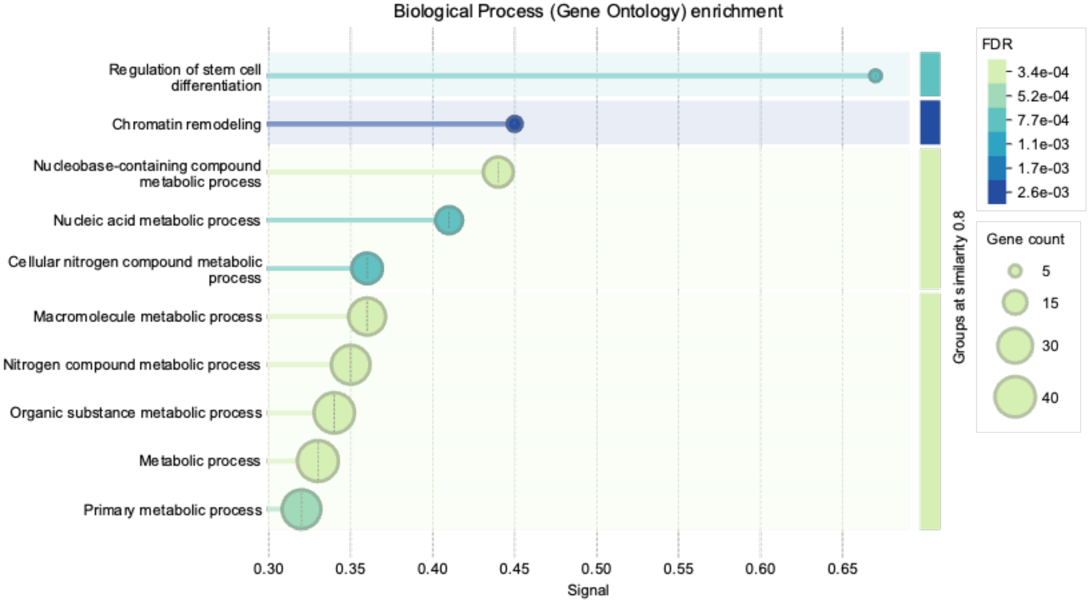

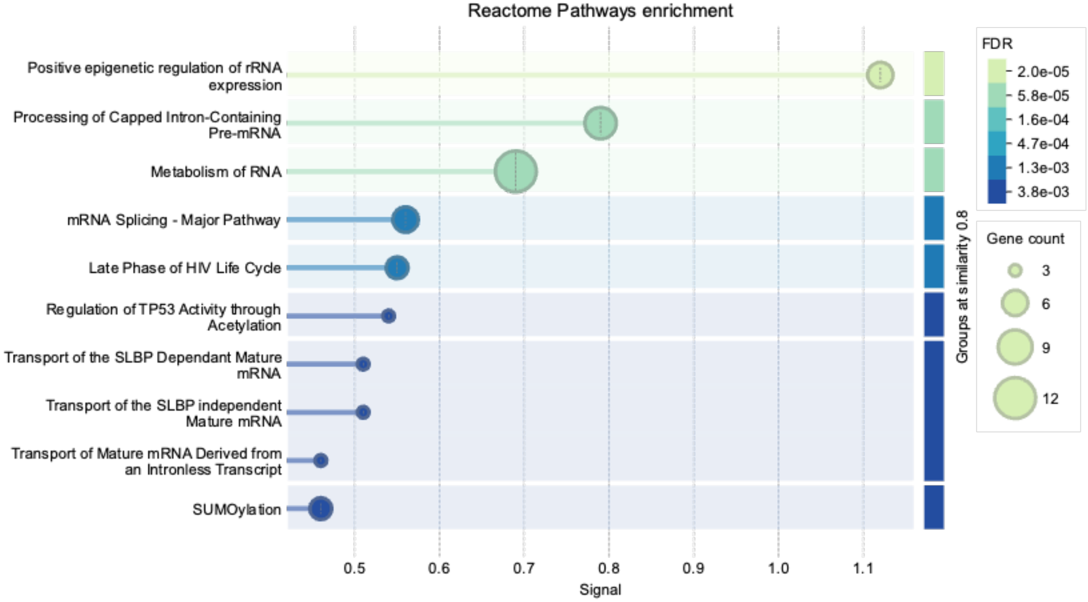

Figure S4. **Biological processes identified** by network enrichment analysis using Gene Ontology (GO) and STRING platforms of 64 adrenocortical carcinomas (ACC) upregulated proteins relative to adrenocortical adenomas (ACA). FDR = false discovery rate.

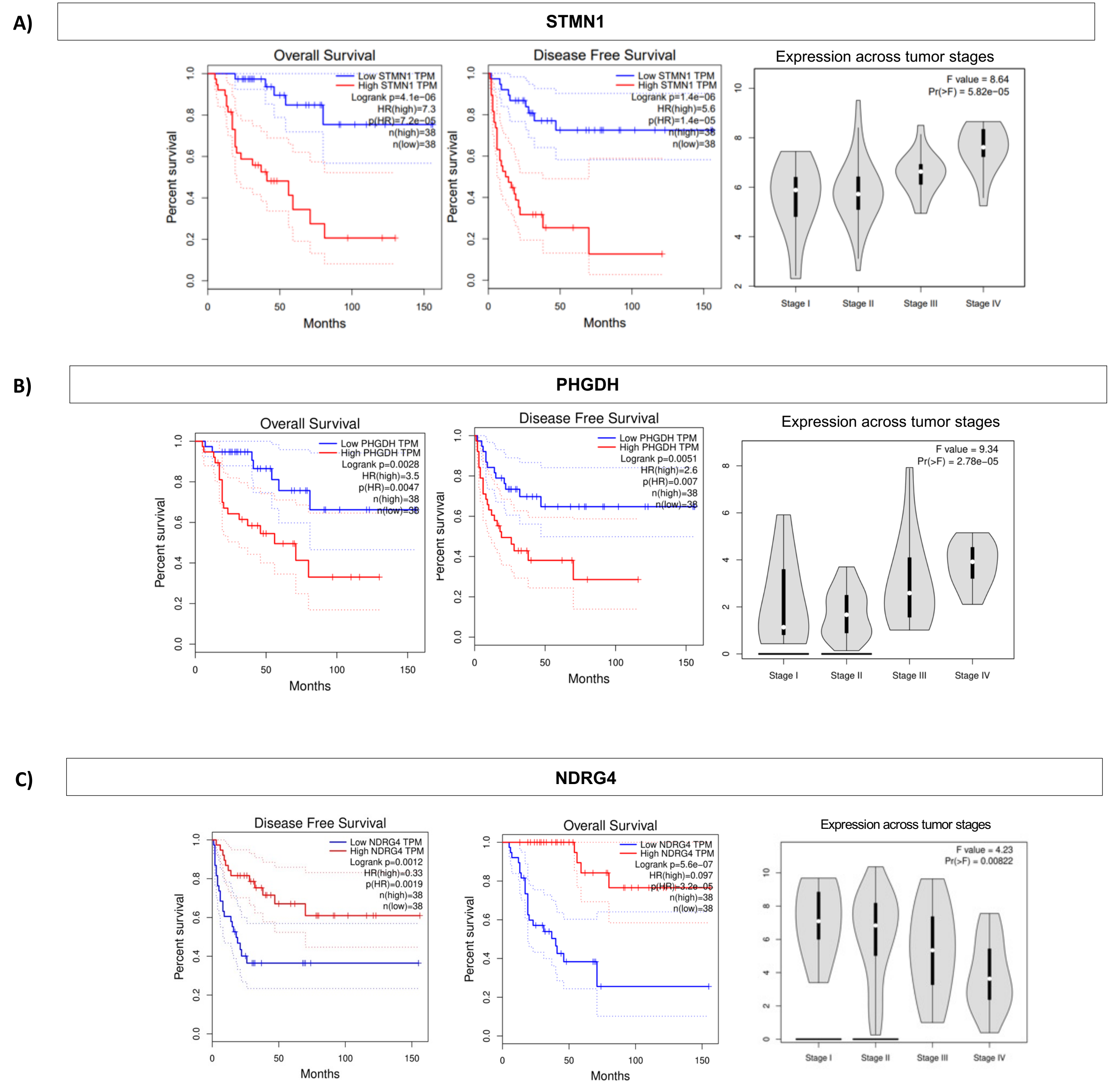

Figure S5. High expression of STMN1 and PHGDH is associated with aggressive behavior and poor patient outcomes in ACC, whereas NDRG4 expression is the opposite. Kaplan–Meier survival plot of overall survival (OS), disease-free survival (DFS), and expression across tumor stages of A) Stathmin 1 (STMN1); B) phosphoglycerate dehydrogenase (PHGDH); C) N-myc downregulated gene family 4 (NDRG4) expression from TCGA analysis. n = 38.
